# Supplementary material for: Performance of phenomic selection in rice: Effects of population size and genotype-environment interactions on predictive ability
Source: PLoS One. 2024 Dec 23;19(12):e0309502. doi: 10.1371/journal.pone.0309502 (PMC11666020; doi:10.1371/journal.pone.0309502)
Supplement: S1 Table — (PDF) [file pone.0309502.s001.pdf]

S1 Table: Descriptive statistics of the phenotypic measurements performed in each of the four environments (2015-HN, 2015-LN, 2016-HN and 2016-LN) with mean, standard deviation (StdDev), min, max and coefficient of variation (CV, %)

Trait[1]	Environment	Mean ± StdDev	Min	Max	CV
DF	15HN	95.6 ± 5.62	79	116	5.86
	15LN	96.7 ± 5.68	81	111	5.87
	16HN	90.4 ± 7.16	70	112	7.92
	16LN	89.4 ± 7.28	70	112	8.15
PH	15HN	108.6 ± 14.6	60.3	160.5	13.4
	15LN	92.7 ± 14.0	49.3	141	15
	16HN	112.1 ± 15.2	66	162.2	13.5
	16LN	96.5 ± 14.2	58.8	146.8	14.7
TGW	15HN	27.2 ± 4.32	14.95	42.1	15.9
	15LN	28.3 ± 4.51	16.9	43.7	15.9
	16HN	28.7 ± 4.60	16.1	44	16
	16LN	28.7 ± 4.54	17.5	42.5	15.8
HI	15HN	0.44 ± 0.08	0.04	0.6	19.5
	15LN	0.48 ± 0.08	0.13	0.64	15.8
	16HN	0.45 ± 0.08	0.15	0.59	16.9
	16LN	0.49 ± 0.06	0.17	0.63	12.4
GY	15HN	4106 ± 1272	227	7345	31
	15LN	3210 ± 1240	504	8080	38.6
	16HN	5359 ± 1412	861	8871	26.4
	16LN	3986 ± 1168	925	7825	29.3
GNC	15HN	1.71 ± 0.21	0.94	2.55	12.6
	15LN	1.29 ± 0.19	0.83	1.93	14.3
	16HN	1.80 ± 0.19	1.28	2.34	10.4
	16LN	1.42 ± 0.20	0.86	2.01	14.2

[1]DF, Date at flowering; PH, plant height; TGW, thousand grain weight; HI, harvest index; GY, grain yield; GNC, grain nitrogen content
