# Supplementary material for: Performance of phenomic selection in rice: Effects of population size and genotype-environment interactions on predictive ability
Source: PLoS One. 2024 Dec 23;19(12):e0309502. doi: 10.1371/journal.pone.0309502 (PMC11666020; doi:10.1371/journal.pone.0309502)
Supplement: S2 Table — (PDF) [file pone.0309502.s002.pdf]

**S2 Table: Results of the LMM analyses comparing all the effects for all the scenarios and all the traits, using Z-transformed PA.**

With one environment – p-values of the effects

| Trait | DF | PH | HI | TGW | GY | GNC |
|---|---|---|---|---|---|---|
| Matrix | **<0.0001** | **<0.0001** | **<0.0001** | **<0.0001** | **<0.0001** | **<0.0001** |
| %TP | 0.0583 | **0.0355** | **0.0079** | **<0.0001** | 0.3605 | **0.0227** |
| Environment | **<0.0001** | **<0.0001** | **<0.0001** | 0.5323 | **<0.0001** | **<0.0001** |
| Matrix*%TP | 0.384 | 0.5485 | 0.8511 | **0.0171** | 0.8459 | 0.4 |
| Matrix*Environment | **0.0046** | 0.1169 | 0.6443 | 0.3131 | **<0.0001** | **<0.0001** |
| %TP*Environment | 0.2105 | 0.1065 | 0.0895 | 0.8824 | 0.1806 | **0.0414** |
| Matrix*%TP*Environment | 0.665 | 0.7046 | 0.9445 | 0.82 | 0.9476 | 0.3231 |

With two environments – p-values of the effects

| Trait | DF | PH | HI | TGW | GY | GNC |
|---|---|---|---|---|---|---|
| Matrix | **<0.0001** | **<0.0001** | **0.0001** | **<0.0001** | **<0.0001** | **<0.0001** |
| %TP | **<0.0001** | **<0.0001** | **<0.0001** | **<0.0001** | **<0.0001** | **<0.0001** |
| Combination of Environments | **<0.0001** | **<0.0001** | **<0.0001** | **<0.0001** | **<0.0001** | **<0.0001** |
| Model (MM or MDs) | **<0.0001** | **0.0004** | **<0.0001** | 0.2458 | **<0.0001** | **<0.0001** |
| Model*Matrix | **<0.0001** | 0.2256 | 0.8513 | 0.4399 | 0.2064 | **<0.0001** |
| Model*%TP | **<0.0001** | **<0.0001** | **<0.0001** | **<0.0001** | **<0.0001** | **<0.0001** |
| Model*Environments | **0.006** | **0.0001** | **<0.0001** | **<0.0001** | **<0.0001** | **<0.0001** |
| Matrix*%TP | **<0.0001** | **<0.0001** | **<0.0001** | **<0.0001** | **<0.0001** | **<0.0001** |
| Matrix*Environments | **0.0209** | **0.0279** | 0.4206 | **0.0118** | 0.0004 | **<0.0001** |
| %TP*Environments | **<0.0001** | **0.0026** | **<0.0001** | **0.0095** | **<0.0001** | **<0.0001** |
| Model*Matrix*%TP | **0.0021** | **0.0009** | **<0.0001** | **0.0005** | **<0.0001** | **<0.0001** |
| Model*Matrix*Environments | 0.8295 | 0.9555 | 0.8799 | 0.5316 | 0.2449 | **<0.0001** |
| Matrix*%TP*Environment | **0.0002** | 0.0946 | **<0.0001** | **<0.0001** | **<0.0001** | **<0.0001** |
| Model*Matrix*%TP*Environments | 0.9936 | 0.939 | 0.999 | 0.9263 | **0.0028** | **<0.0001** |

With three environments – p-values of the effects

| Trait | DF | PH | HI | TGW | GY | GNC |
|---|---|---|---|---|---|---|
| Matrix | **<0.0001** | **<0.0001** | 0.388 | **<0.0001** | 0.1759 | **<0.0001** |
| Combination of Environments | **<0.0001** | **<0.0001** | **<0.0001** | **<0.0001** | **<0.0001** | **0.0052** |
| Matrix*Environments | 0.4661 | **0.0053** | 0.974 | 0.0757 | 0.3158 | 0.4566 |
