# Supplementary material for: Performance of phenomic selection in rice: Effects of population size and genotype-environment interactions on predictive ability
Source: PLoS One. 2024 Dec 23;19(12):e0309502. doi: 10.1371/journal.pone.0309502 (PMC11666020; doi:10.1371/journal.pone.0309502)
Supplement: S3 Table — (PDF) [file pone.0309502.s003.pdf]

S3 Table: Predictive ability of the different scenarios and models (means ± standard deviation).

For each trait and effect, letters indicated significantly different effects.

With one environment

Main effect	Matrix	%TP	Environment	LSmean ± SE					
				DF	PH	HI	TGW	GY	GNC
Matrix	GP			0.241 ± 0.0175b	0.7961 ± 0.0289a	0.268 ± 0.0159a	0.8971 ± 0.0305a	0.3061 ± 0.0153a	0.356 ± 0.0678c
	PP			0.143 ± 0.0175c	0.0602 ± 0.0289b	0.112 ± 0.0159b	0.0977 ± 0.0305b	0.0814 ± 0.0153b	2.317 ± 0.0678b
	GP+PP			0.362 ± 0.0175a	0.796 ± 0.0289a	0.306 ± 0.0159a	0.9755 ± 0.0305a	0.3374 ± 0.0153a	3.14 ± 0.0678a
%TP		50%		0.229 ± 0.0143	0.515 ± 0.0236b	0.204 ± 0.0129b	0.571 ± 0.0249b	0.234 ± 0.0125	1.85 ± 0.0554b
		80%		0.268 ± 0.0143	0.586 ± 0.0236a	0.253 ± 0.0129a	0.743 ± 0.0249a	0.25 ± 0.0125	2.03 ± 0.0554a
Environment			15HN	0.198 ± 0.0202b	0.737 ± 0.0334a	0.262 ± 0.0183ab	0.698 ± 0.0353	0.269 ± 0.0177a	2.596 ± 0.0783a
			16HN	0.228 ± 0.0202b	0.578 ± 0.0334b	0.282 ± 0.0183a	0.651 ± 0.0353	0.325 ± 0.0177a	2.73 ± 0.0783a
			15LN	0.231 ± 0.0202b	0.383 ± 0.0334c	0.201 ± 0.0183bc	0.654 ± 0.0353	0.267 ± 0.0177a	0.897 ± 0.0783c
			16LN	0.337 ± 0.0202a	0.505 ± 0.0334bc	0.168 ± 0.0183c	0.625 ± 0.0353	0.106 ± 0.0177b	1.528 ± 0.0783b
Matrix*%TP	PP	2		0.142 ± 0.0247	0.05 ± 0.0409	0.0934 ± 0.0224	0.0721 ± 0.0432c	0.0663 ± 0.0217b	2.169 ± 0.0959
	PP	5		0.144 ± 0.0247	0.0704 ± 0.0409	0.1313 ± 0.0224	0.1233 ± 0.0432c	0.0964 ± 0.0217b	2.465 ± 0.0959
	GP	2		0.219 ± 0.0247	0.7535 ± 0.0409	0.2361 ± 0.0224	0.8147 ± 0.0432b	0.2997 ± 0.0217a	0.33 ± 0.0959
	GP	5		0.262 ± 0.0247	0.8387 ± 0.0409	0.2991 ± 0.0224	0.9795 ± 0.0432ab	0.3126 ± 0.0217a	0.383 ± 0.0959
	GPPP	2		0.327 ± 0.0247	0.7428 ± 0.0409	0.2824 ± 0.0224	0.8256 ± 0.0432b	0.3346 ± 0.0217a	3.045 ± 0.0959
	GPPP	5		0.397 ± 0.0247	0.8493 ± 0.0409	0.3288 ± 0.0224	1.1254 ± 0.0432a	0.3403 ± 0.0217a	3.235 ± 0.0959
Matrix*Environment	PP		16HN	0.0366 ± 0.035e	0.0715 ± 0.0578	0.1464 ± 0.0317	0.0636 ± 0.0611	0.2339 ± 0.0307cde	2.742 ± 0.136c
	PP		15LN	0.1482 ± 0.035de	-0.0153 ± 0.0578	0.0703 ± 0.0317	0.0567 ± 0.0611	0.0249 ± 0.0307f	1.024 ± 0.136ef
	PP		15HN	0.1682 ± 0.035cde	0.1369 ± 0.0578	0.1467 ± 0.0317	0.2107 ± 0.0611	0.0961 ± 0.0307ef	3.778 ± 0.136b
	GP		15HN	0.1685 ± 0.035cde	1.0304 ± 0.0578	0.2884 ± 0.0317	0.8451 ± 0.0611	0.3592 ± 0.0307abc	0.29 ± 0.136g
	PP		16LN	0.2191 ± 0.035bcd	0.0477 ± 0.0578	0.0859 ± 0.0317	0.0596 ± 0.0611	-0.0293 ± 0.0307f	1.722 ± 0.136d
	GP		15LN	0.2201 ± 0.035bcd	0.5721 ± 0.0578	0.2514 ± 0.0317	0.9192 ± 0.0611	0.3933 ± 0.0307ab	0.342 ± 0.136g
	GPPP		15HN	0.2575 ± 0.035bcd	1.0424 ± 0.0578	0.3519 ± 0.0317	1.0371 ± 0.0611	0.3505 ± 0.0307abc	3.718 ± 0.136b
	GP		16HN	0.2718 ± 0.035bcd	0.8586 ± 0.0578	0.3529 ± 0.0317	0.9701 ± 0.0611	0.296 ± 0.0307bcd	0.438 ± 0.136fg
	GP		16LN	0.3028 ± 0.035bcd	0.7232 ± 0.0578	0.1777 ± 0.0317	0.854 ± 0.0611	0.176 ± 0.0307de	0.356 ± 0.136g
	GPPP		15LN	0.3233 ± 0.035bcd	0.5931 ± 0.0578	0.2822 ± 0.0317	0.9858 ± 0.0611	0.3828 ± 0.0307ab	1.325 ± 0.136de
	GPPP		16HN	0.3766 ± 0.035ab	0.805 ± 0.0578	0.3465 ± 0.0317	0.9185 ± 0.0611	0.4437 ± 0.0307a	5.011 ± 0.136a
	GPPP		16LN	0.4898 ± 0.035a	0.7436 ± 0.0578	0.2417 ± 0.0317	0.9606 ± 0.0611	0.1728 ± 0.0307de	2.506 ± 0.136c
%TP*Environment		5	15HN	0.195 ± 0.0286	0.836 ± 0.0472	0.308 ± 0.0259	0.789 ± 0.0499	0.307 ± 0.025	2.845 ± 0.111a
		2	15HN	0.201 ± 0.0286	0.637 ± 0.0472	0.216 ± 0.0259	0.606 ± 0.0499	0.23 ± 0.025	2.346 ± 0.111b
		2	16HN	0.212 ± 0.0286	0.538 ± 0.0472	0.233 ± 0.0259	0.556 ± 0.0499	0.318 ± 0.025	2.603 ± 0.111ab
		2	15LN	0.221 ± 0.0286	0.372 ± 0.0472	0.207 ± 0.0259	0.557 ± 0.0499	0.283 ± 0.025	0.956 ± 0.111d
		5	15LN	0.24 ± 0.0286	0.395 ± 0.0472	0.196 ± 0.0259	0.751 ± 0.0499	0.251 ± 0.025	0.838 ± 0.111d
		5	16HN	0.244 ± 0.0286	0.619 ± 0.0472	0.331 ± 0.0259	0.745 ± 0.0499	0.331 ± 0.025	2.857 ± 0.111a
		2	16LN	0.283 ± 0.0286	0.516 ± 0.0472	0.159 ± 0.0259	0.563 ± 0.0499	0.104 ± 0.025	1.486 ± 0.111c
		5	16LN	0.392 ± 0.0286	0.494 ± 0.0472	0.178 ± 0.0259	0.686 ± 0.0499	0.109 ± 0.025	1.57 ± 0.111c
Matrix*%TP*Environment	PP	5	16HN	0.0235 ± 0.0495	0.08537 ± 0.0818	0.2036 ± 0.0449	0.0849 ± 0.0864	0.2696 ± 0.0434	2.875 ± 0.192
	PP	2	16HN	0.0498 ± 0.0495	0.0576 ± 0.0818	0.0893 ± 0.0449	0.0423 ± 0.0864	0.1981 ± 0.0434	2.61 ± 0.192
	PP	5	15LN	0.1458 ± 0.0495	0.00174 ± 0.0818	0.0416 ± 0.0449	0.0713 ± 0.0864	0.0103 ± 0.0434	0.93 ± 0.192
	PP	2	15LN	0.1505 ± 0.0495	-0.03226 ± 0.0818	0.099 ± 0.0449	0.042 ± 0.0864	0.0394 ± 0.0434	1.119 ± 0.192
	PP	2	15HN	0.1541 ± 0.0495	0.12501 ± 0.0818	0.1132 ± 0.0449	0.154 ± 0.0864	0.0629 ± 0.0434	3.368 ± 0.192
	GP	5	15HN	0.1608 ± 0.0495	1.14991 ± 0.0818	0.3332 ± 0.0449	0.8996 ± 0.0864	0.3948 ± 0.0434	0.276 ± 0.192
	GP	2	15HN	0.1763 ± 0.0495	0.91082 ± 0.0818	0.2437 ± 0.0449	0.7905 ± 0.0864	0.3236 ± 0.0434	0.303 ± 0.192
	PP	5	15HN	0.1824 ± 0.0495	0.14875 ± 0.0818	0.1801 ± 0.0449	0.2675 ± 0.0864	0.1293 ± 0.0434	4.188 ± 0.192
	GP	2	15LN	0.202 ± 0.0495	0.54534 ± 0.0818	0.23 ± 0.0449	0.7811 ± 0.0864	0.3986 ± 0.0434	0.291 ± 0.192
	PP	2	16LN	0.2137 ± 0.0495	0.04981 ± 0.0818	0.0719 ± 0.0449	0.0499 ± 0.0864	-0.0351 ± 0.0434	1.579 ± 0.192
	PP	5	16LN	0.2246 ± 0.0495	0.04555 ± 0.0818	0.1 ± 0.0449	0.0693 ± 0.0864	-0.0235 ± 0.0434	1.866 ± 0.192
	GP	5	15LN	0.2383 ± 0.0495	0.59892 ± 0.0818	0.2728 ± 0.0449	1.0573 ± 0.0864	0.3879 ± 0.0434	0.393 ± 0.192
	GPPP	5	15HN	0.241 ± 0.0495	1.21076 ± 0.0818	0.4121 ± 0.0449	1.2004 ± 0.0864	0.3982 ± 0.0434	4.07 ± 0.192
	GP	2	16HN	0.2462 ± 0.0495	0.83325 ± 0.0818	0.3107 ± 0.0449	0.9166 ± 0.0864	0.2946 ± 0.0434	0.401 ± 0.192
	GP	2	16LN	0.2527 ± 0.0495	0.72465 ± 0.0818	0.1601 ± 0.0449	0.7705 ± 0.0864	0.1819 ± 0.0434	0.323 ± 0.192
	GPPP	2	15HN	0.2741 ± 0.0495	0.87401 ± 0.0818	0.2917 ± 0.0449	0.8738 ± 0.0864	0.3027 ± 0.0434	3.367 ± 0.192
	GP	5	16HN	0.2974 ± 0.0495	0.88399 ± 0.0818	0.3952 ± 0.0449	1.0237 ± 0.0864	0.2974 ± 0.0434	0.475 ± 0.192
	GPPP	2	15LN	0.3099 ± 0.0495	0.6026 ± 0.0818	0.2923 ± 0.0449	0.8485 ± 0.0864	0.4098 ± 0.0434	1.459 ± 0.192
	GPPP	5	15LN	0.3366 ± 0.0495	0.58368 ± 0.0818	0.2721 ± 0.0449	1.1231 ± 0.0864	0.3558 ± 0.0434	1.191 ± 0.192
	GPPP	2	16HN	0.341 ± 0.0495	0.72254 ± 0.0818	0.2996 ± 0.0449	0.7103 ± 0.0864	0.4602 ± 0.0434	4.798 ± 0.192
	GP	5	16LN	0.353 ± 0.0495	0.72184 ± 0.0818	0.1953 ± 0.0449	0.9375 ± 0.0864	0.1701 ± 0.0434	0.389 ± 0.192
	GPPP	2	16LN	0.3816 ± 0.0495	0.77209 ± 0.0818	0.2459 ± 0.0449	0.8697 ± 0.0864	0.1657 ± 0.0434	2.557 ± 0.192
	GPPP	5	16HN	0.4122 ± 0.0495	0.88745 ± 0.0818	0.3934 ± 0.0449	1.1266 ± 0.0864	0.4271 ± 0.0434	5.223 ± 0.192
	GPPP	5	16LN	0.5979 ± 0.0495	0.71517 ± 0.0818	0.2375 ± 0.0449	1.0515 ± 0.0864	0.1799 ± 0.0434	2.455 ± 0.192

With two environments - only single effects are included in the table

Main effect	Effect	DF	PH	HI	TGW	GY	GNC
Matrix	GP	2.73 ± 0.105b	1.75 ± 0.0466b	0.98 ± 0.0171b	2.76 ± 0.0646b	0.499 ± 0.0066a	0.552 ± 0.0233c
	PP	2.8 ± 0.105b	1.61 ± 0.0466b	0.95 ± 0.0171b	2.59 ± 0.0646b	0.474 ± 0.0066b	1.029 ± 0.0233b
	GP+PP	3.42 ± 0.105a	2.02 ± 0.0466a	1.05 ± 0.0171a	3.3 ± 0.0646a	0.518 ± 0.0066a	1.202 ± 0.0233a
% Target env included in the TP	0	1.95 ± 0.117c	1.45 ± 0.0538c	0.777 ± 0.0197c	1.94 ± 0.0746c	0.381 ± 0.00762d	0.408 ± 0.0269c
	0.1	3.8 ± 0.117a	2.15 ± 0.0538a	1.145 ± 0.0197a	3.59 ± 0.0746a	0.58 ± 0.00762a	1.084 ± 0.0269b
	0.5	3 ± 0.117b	1.74 ± 0.0538b	1.018 ± 0.0197b	2.97 ± 0.0746b	0.537 ± 0.00762b	1.018 ± 0.0269b
	0.8	3.18 ± 0.117b	1.84 ± 0.0538b	1.029 ± 0.0197b	3.03 ± 0.0746b	0.49 ± 0.00762c	1.2 ± 0.0269a
Model	MDs	2.69 ± 0.0824b	1.7 ± 0.038b	1.037 ± 0.0139a	2.84 ± 0.0527	0.534 ± 0.00539a	1.282 ± 0.019a
	MM	3.28 ± 0.0824a	1.89 ± 0.038a	0.947 ± 0.0139b	2.92 ± 0.0527	0.46 ± 0.00539b	0.573 ± 0.019b
Combination of Environments	15HN_16HN	1.04 ± 0.143d	2.11 ± 0.0659a	0.8 ± 0.0241d	2.16 ± 0.0913c	0.61 ± 0.00933a	1.332 ± 0.0329a
	15LN_16LN	1.34 ± 0.143d	1.32 ± 0.0659d	0.881 ± 0.0241cd	2 ± 0.0913c	0.465 ± 0.00933c	0.753 ± 0.0329cd
	15LN_15HN	2.84 ± 0.143c	1.79 ± 0.0659bc	1.136 ± 0.0241b	3.23 ± 0.0913ab	0.48 ± 0.00933c	0.932 ± 0.0329b
	15HN_15LN	3.36 ± 0.143c	1.62 ± 0.0659c	1.331 ± 0.0241a	3.34 ± 0.0913ab	0.527 ± 0.00933b	0.673 ± 0.0329d
	16LN_16HN	4.07 ± 0.143b	1.89 ± 0.0659ab	0.855 ± 0.0241cd	3.05 ± 0.0913b	0.453 ± 0.00933c	1.001 ± 0.0329b
	16HN_16LN	5.25 ± 0.143a	2.06 ± 0.0659a	0.95 ± 0.0241c	3.52 ± 0.0913a	0.444 ± 0.00933c	0.875 ± 0.0329bc

With three environments - only single effects are included in the table

Effect	Matrix	Environments	DF	PH	HI	TGW	GY	GNC
Matrix	GPPP		1.79 ± 0.131b	1.85 ± 0.0636b	0.795 ± 0.0362	2.84 ± 0.0877b	0.391 ± 0.0179	1.615 ± 0.0538a
	PP		1.83 ± 0.131b	1.7 ± 0.0636b	0.854 ± 0.0362	2.92 ± 0.0877b	0.381 ± 0.0179	1.764 ± 0.0538a
	GP		2.49 ± 0.131a	2.37 ± 0.0636a	0.858 ± 0.0362	3.49 ± 0.0877a	0.426 ± 0.0179	0.591 ± 0.0538b
Combination of Environments		15HN_15LN_16LN	1.09 ± 0.152b	1.66 ± 0.0734b	0.618 ± 0.0419b	2.17 ± 0.101c	0.27 ± 0.0207c	1.16 ± 0.0622b
		15HN_15LN_16HN	1.17 ± 0.152b	1.37 ± 0.0734c	0.717 ± 0.0419b	2.14 ± 0.101c	0.45 ± 0.0207ab	1.31 ± 0.0622ab
		15HN_16LN_16HN	2.72 ± 0.152a	2.45 ± 0.0734a	0.953 ± 0.0419a	4.37 ± 0.101a	0.499 ± 0.0207a	1.48 ± 0.0622a
		15LN_16HN_16LN	3.17 ± 0.152a	2.4 ± 0.0734a	1.055 ± 0.0419a	3.65 ± 0.101b	0.38 ± 0.0207b	1.35 ± 0.0622ab
Matrix*Combination of Environments	GPPP	15HN_15LN_16HN	0.984 ± 0.263	1.188 ± 0.127e	0.693 ± 0.0725	1.91 ± 0.175	0.452 ± 0.0358	1.545 ± 0.108
	GPPP	15HN_15LN_16LN	0.996 ± 0.263	1.554 ± 0.127cde	0.589 ± 0.0725	2 ± 0.175	0.248 ± 0.0358	1.497 ± 0.108
	PP	15HN_15LN_16LN	0.997 ± 0.263	1.455 ± 0.127de	0.668 ± 0.0725	1.93 ± 0.175	0.292 ± 0.0358	1.557 ± 0.108
	PP	15HN_15LN_16HN	1.009 ± 0.263	0.966 ± 0.127e	0.711 ± 0.0725	1.79 ± 0.175	0.421 ± 0.0358	1.697 ± 0.108
	GP	15HN_15LN_16LN	1.281 ± 0.263	1.968 ± 0.127bcd	0.598 ± 0.0725	2.57 ± 0.175	0.271 ± 0.0358	0.419 ± 0.108
	GP	15HN_15LN_16HN	1.508 ± 0.263	1.961 ± 0.127bcd	0.746 ± 0.0725	2.72 ± 0.175	0.477 ± 0.0358	0.682 ± 0.108
	GPPP	15HN_16LN_16HN	2.256 ± 0.263	2.088 ± 0.127bc	0.891 ± 0.0725	3.99 ± 0.175	0.515 ± 0.0358	1.663 ± 0.108
	PP	15HN_16LN_16HN	2.318 ± 0.263	2.235 ± 0.127b	0.951 ± 0.0725	4.15 ± 0.175	0.423 ± 0.0358	2.028 ± 0.108
	GPPP	15LN_16HN_16LN	2.916 ± 0.263	2.551 ± 0.127ab	1.007 ± 0.0725	3.45 ± 0.175	0.349 ± 0.0358	1.754 ± 0.108
	PP	15LN_16HN_16LN	3.014 ± 0.263	2.147 ± 0.127bc	1.085 ± 0.0725	3.8 ± 0.175	0.39 ± 0.0358	1.774 ± 0.108
	GP	15LN_16HN_16LN	3.595 ± 0.263	2.513 ± 0.127ab	1.073 ± 0.0725	3.7 ± 0.175	0.4 ± 0.0358	0.521 ± 0.108
	GP	15HN_16LN_16HN	3.596 ± 0.263	3.022 ± 0.127a	1.016 ± 0.0725	4.97 ± 0.175	0.558 ± 0.0358	0.742 ± 0.108
