# Supplementary material for: Performance of phenomic selection in rice: Effects of population size and genotype-environment interactions on predictive ability
Source: PLoS One. 2024 Dec 23;19(12):e0309502. doi: 10.1371/journal.pone.0309502 (PMC11666020; doi:10.1371/journal.pone.0309502)
Supplement: S4 Table — (PDF) [file pone.0309502.s004.pdf]

S4 Table: List of the rice accessions used

Genotype name / accession name	Country	Breeding center	Panel ID
126-C409-8-1-2	Colombia	CIAT	1
B22	Brazil	EMBRAPA	2
C537B	Madagascar	FOFIFA-Cirad	3
C507 1373-1-b-2--	Madagascar	FOFIFA-Cirad	4
C630 139-46-2-3-3-b-1-1-1	Madagascar	FOFIFA-Cirad	5
C630 38-4-1-b-3-2-1-b-b	Madagascar	FOFIFA-Cirad	6
Caiapo	Brazil		8
Cha Loy Oe	Thailand		9
Chhomrong Dhan	Nepal		10
Cirad 141	Brazil	Cirad	11
Cirad 392	Madagascar	FOFIFA-Cirad	12
Cirad 394	Madagascar	FOFIFA-Cirad	13
Cirad 409	Brazil	Cirad	15
Cirad 447	Brazil	Cirad	16
Cirad 488	Madagascar	FOFIFA-Cirad	17
CNA 4123	Brazil	EMBRAPA	18
CNA 4136	Brazil	EMBRAPA	19
CNA 4137	Brazil	EMBRAPA	20
CNA 4196	Brazil	EMBRAPA	21
CNA-IREM 190	Brazil	EMBRAPA	22
CT13582-15-5-M	Colombia	CIAT	23
Cuiabana	Brazil		24
Curinca	Brazil		25
Dangrey	Bhutan		26
Daniela	Brazil		27
Dourado Precoce	Brazil		28
Early Mutant IAC165	Brazil		29
Estrela	Brazil		31
EXP 003	Madagascar	FOFIFA-Cirad	32
EXP 006	Madagascar	FOFIFA-Cirad	33
EXP 011	Madagascar	FOFIFA-Cirad	34
EXP 013	Madagascar	FOFIFA-Cirad	35
EXP 202	Madagascar	FOFIFA-Cirad	36
EXP 206	Madagascar	FOFIFA-Cirad	37
EXP 302	Madagascar	FOFIFA-Cirad	38
EXP 303	Madagascar	FOFIFA-Cirad	39
EXP 304	Madagascar	FOFIFA-Cirad	40
EXP 401	Madagascar	FOFIFA-Cirad	41
EXP 409	Madagascar	FOFIFA-Cirad	42
EXP 910	Madagascar	FOFIFA-Cirad	43
F152.06.33.53 13-1-5-1-1	Madagascar	FOFIFA-Cirad	44

F154.3G.04.121.10 1	Madagascar	FOFIFA-Cirad	46
FOFIFA 116	Madagascar	FOFIFA-Cirad	47
FOFIFA 151	Madagascar	FOFIFA-Cirad	48
FOFIFA 159	Madagascar	FOFIFA-Cirad	49
FOFIFA 167	Madagascar	FOFIFA-Cirad	50
FOFIFA 168	Madagascar	FOFIFA-Cirad	51
FOFIFA 171	Madagascar	FOFIFA-Cirad	52
FOFIFA 172	Madagascar	FOFIFA-Cirad	53
FOFIFA 173	Madagascar	FOFIFA-Cirad	54
FOFIFA 180	Madagascar	FOFIFA-Cirad	55
FOFIFA 181	Madagascar	FOFIFA-Cirad	56
FOFIFA 62	Madagascar	FOFIFA-Cirad	57
Guarani	Brazil		58
HD 1-4	France		59
IAC 1205	Brazil	IAC	60
IAC 25	Brazil	IAC	61
IR 53236-275-1	Philippines	IRRI	62
IR 66421-105-1-1	Philippines	IRRI	63
IRAT 109	Brazil	Cirad	64
IRAT 112	Brazil	Cirad	65
IRAT 13	Brazil	Cirad	66
IRAT 134	Brazil	Cirad	67
IRAT 212	Brazil	Cirad	68
IRAT 234	Brazil	Cirad	69
IRAT 265	Brazil	Cirad	70
IRAT 367	Brazil	Cirad	71
IRAT 380	Brazil	Cirad	72
IREM 239	Brazil		73
Kuroka	Japan		74
Luluwini 22M	Brazil		75
Munumliguero	Brazil		76
Nabeshi	Taiwan		77
Nerica 1	Ivory Coast	AfricaRice	78
Nerica 10	Ivory Coast	AfricaRice	79
Nerica 11	Ivory Coast	AfricaRice	80
Nerica 12	Ivory Coast	AfricaRice	81
Nerica 13	Ivory Coast	AfricaRice	82
Nerica 16	Ivory Coast	AfricaRice	83
Nerica 18	Ivory Coast	AfricaRice	84
Nerica 2	Ivory Coast	AfricaRice	85
Nerica 3	Ivory Coast	AfricaRice	86
Nerica 4	Ivory Coast	AfricaRice	197
Nerica 5	Ivory Coast	AfricaRice	87
Nerica 6	Ivory Coast	AfricaRice	88
Nerica 7	Ivory Coast	AfricaRice	89

Nerica 8	Ivory Coast	AfricaRice	90
Nerica 9	Ivory Coast	AfricaRice	91
PCT11 MAD2007\0\0 14-1-1-1-3-3-2	Colombia	CIAT	92
PCT11 MAD2007\0\0 28-3-3-5-5-5	Colombia	CIAT	93
PCT11 MAD2007\0\0 3-3-1-3-2-2-4	Colombia	CIAT	94
PCT11 MAD2007\0\0 3-5-5-2-1-4-4	Colombia	CIAT	95
PCT11 MAD2007\0\0 50-1-1-1-5-5-5	Colombia	CIAT	96
PCT11 x CNA7 42-3-2	Colombia	CIAT	97
PCT11 x CNA7 73-2-5	Colombia	CIAT	98
PCT11\0\0\2\Bo\2\1>181	Colombia	CIAT	99
PCT-4 MAD2007\0\1 18-2--1-5-2-3	Colombia	CIAT	100
PCT-4\0\0\1>5-M-1-6	Colombia	CIAT	101
PCT-4\SA\1\1\SA\2\1>746-1-5-4-1 5-5-1-1-1	Colombia	CIAT	102
PCT-4\SA\1\1>975-M-2-M-3 2-5-5-1-1	Colombia	CIAT	103
PCT-4\SA\4\1>1076-2-4-1-5	Colombia	CIAT	104
PCT-4\SA\4\1>330-1-4-5-1-M 1-1-1-1-2	Colombia	CIAT	105
PCT-4\SA\4\1>330-2-2-3-2-M 5-4-4-3-1-5	Colombia	CIAT	106
PCT-5\PHB\1\0.PHB\1.PHB\1.PHB\1>78-2--6-2-M	Colombia	CIAT	107
Primavera	Brazil	IAC	108
SCRID036 4-1-1-5-M	Madagascar	FOFIFA-Cirad	109
SCRID090 148-1-2-4-5-4-2	Madagascar	FOFIFA-Cirad	110
SCRID090 60-162-3-4-1-2	Madagascar	FOFIFA-Cirad	111
SCRID090 72-3-1-3-5-1--	Madagascar	FOFIFA-Cirad	112
SCRID090 89-1-5-4-2-2	Madagascar	FOFIFA-Cirad	113
SCRID091 10-1-3-2-5-3-2	Madagascar	FOFIFA-Cirad	114
SCRID 091 11-1-4-3-2-4-3	Madagascar	FOFIFA-Cirad	115
SCRID091 15-2-2-1-1-2	Madagascar	FOFIFA-Cirad	116
SCRID091 24-3-2-2-3-5-4	Madagascar	FOFIFA-Cirad	117
SCRID111 1-4-3-3-5-5-4	Madagascar	FOFIFA-Cirad	118
SCRID128 1-3-4-2-4-4	Madagascar	FOFIFA-Cirad	119
SCRID 128 18-5-4-4-5-3	Madagascar	FOFIFA-Cirad	120
SCRID128 21-3-1-1-1-3	Madagascar	FOFIFA-Cirad	122
SCRID136 20-1-1-1	Madagascar	FOFIFA-Cirad	123
SCRID139 18-2-4-1-1-3-1	Madagascar	FOFIFA-Cirad	124
SCRID139 9-1-5-2-4-4-1	Madagascar	FOFIFA-Cirad	125
SCRID195 11-4-4-2-4-3	Madagascar	FOFIFA-Cirad	127
SCRID195 67-1-1-2-2	Madagascar	FOFIFA-Cirad	129
SCRID195 A1-3-4-2-4-3	Madagascar	FOFIFA-Cirad	130
SCRID195-1-5-3	Madagascar	FOFIFA-Cirad	131
SCRID200 15-4-2-4-1	Madagascar	FOFIFA-Cirad	132
SCRID222 122-4-3-3	Madagascar	FOFIFA-Cirad	133
SCRID222 134-1-1-2	Madagascar	FOFIFA-Cirad	134
SCRID222 164-1-1-4	Madagascar	FOFIFA-Cirad	135
SCRID241 1-1-1-1	Madagascar	FOFIFA-Cirad	136
SCRID242 22-1-2	Madagascar	FOFIFA-Cirad	137

SCRID243 52-1-1-4	Madagascar	FOFIFA-Cirad	138
SCRID251 25-2-1-2	Madagascar	FOFIFA-Cirad	139
SCRID251 95-1-1-3	Madagascar	FOFIFA-Cirad	140
SCRID252 18-1-2-4	Madagascar	FOFIFA-Cirad	141
SCRID253 5-2-2-2	Madagascar	FOFIFA-Cirad	142
SCRID254 85-3-2-3	Madagascar	FOFIFA-Cirad	143
SCRID260 19-2-1-2	Madagascar	FOFIFA-Cirad	144
SCRID264 69-1-2	Madagascar	FOFIFA-Cirad	145
SCRID271 12-1-3	Madagascar	FOFIFA-Cirad	146
SCRID271 37-1-1	Madagascar	FOFIFA-Cirad	147
SCRID271 67-3-3	Madagascar	FOFIFA-Cirad	148
SCRID273 17-1-2	Madagascar	FOFIFA-Cirad	149
SCRID273 25-1-3	Madagascar	FOFIFA-Cirad	150
SCRID274 30-2-3	Madagascar	FOFIFA-Cirad	151
SCRID275 13-1-5	Madagascar	FOFIFA-Cirad	152
SCRID275-72-5-5	Madagascar	FOFIFA-Cirad	153
SCRID278-148-5-1	Madagascar	FOFIFA-Cirad	154
SCRID278 151-5-1	Madagascar	FOFIFA-Cirad	155
SCRID278 42-2-3	Madagascar	FOFIFA-Cirad	156
SCRID292 116-4-2	Madagascar	FOFIFA-Cirad	157
SCRID292 24-2-5	Madagascar	FOFIFA-Cirad	158
SCRID6 4-3-M	Madagascar	FOFIFA-Cirad	159
Sebota 239	Brazil	Cirad	160
Sebota 33	Brazil	Cirad	161
Sebota 330	Brazil	Cirad	162
Sebota 400	Brazil	Cirad	164
Sebota 401	Brazil	Cirad	165
Sebota 402	Brazil	Cirad	166
Sebota 403	Brazil	Cirad	167
Sebota 404	Brazil	Cirad	168
Sebota 405	Brazil	Cirad	169
Sebota 406	Brazil	Cirad	170
Sebota 408	Brazil	Cirad	171
Sebota 409	Brazil	Cirad	172
Sebota 410	Brazil	Cirad	173
Sebota 65	Brazil	Cirad	174
Sebota 70	Brazil	Cirad	175
Sebota 337	Brazil	Cirad	176
Sucupira	Brazil		177
Tres Meses	Brazil		178
WAB 450-11-1-P28-1-HB	Ivory Coast	AfricaRice	179
WAB 450-25-2-9-4-1-B-HB	Ivory Coast	AfricaRice	180
WAB 56-125	Ivory Coast	AfricaRice	181
WAB 56-50	Ivory Coast	AfricaRice	182
WAB 706-3-4-K4-KB-1	Ivory Coast	AfricaRice	183

WAB 758-1-1-HB-4	Ivory Coast	AfricaRice	184
WAB 759-54-2-3-HB-2B	Ivory Coast	AfricaRice	185
WAB 775-95-2-2-HB-1/Cirad 409-3 1-2-5-3-1	Ivory Coast	AfricaRice	186
WAB 788-18-2-2-HB-2/PCT-4\SA\1\1>721-M-2-M-4-M-2-M-5-M-1	Ivory Coast	AfricaRice	187
WAB 878-6-12-1-1-P1-HB	Ivory Coast	AfricaRice	188
WAB 880-1-32-1-1-P2-HB-1-1-2-2	Ivory Coast	AfricaRice	198
WAB 891SG26	Ivory Coast	AfricaRice	189
WAB 891SG9	Ivory Coast	AfricaRice	190
Yangkum red	Bhutan		191
Yunlu 48	China	YAAS	196
Yunlu 50	China	YAAS	195
Yunlu 64	China	YAAS	192
Yunlu 65	China	YAAS	193
Yunlu 7	China	YAAS	194
