# Supplementary material for: Performance of phenomic selection in rice: Effects of population size and genotype-environment interactions on predictive ability
Source: PLoS One. 2024 Dec 23;19(12):e0309502. doi: 10.1371/journal.pone.0309502 (PMC11666020; doi:10.1371/journal.pone.0309502)
Supplement: S1 Fig — (PDF) [file pone.0309502.s005.pdf]

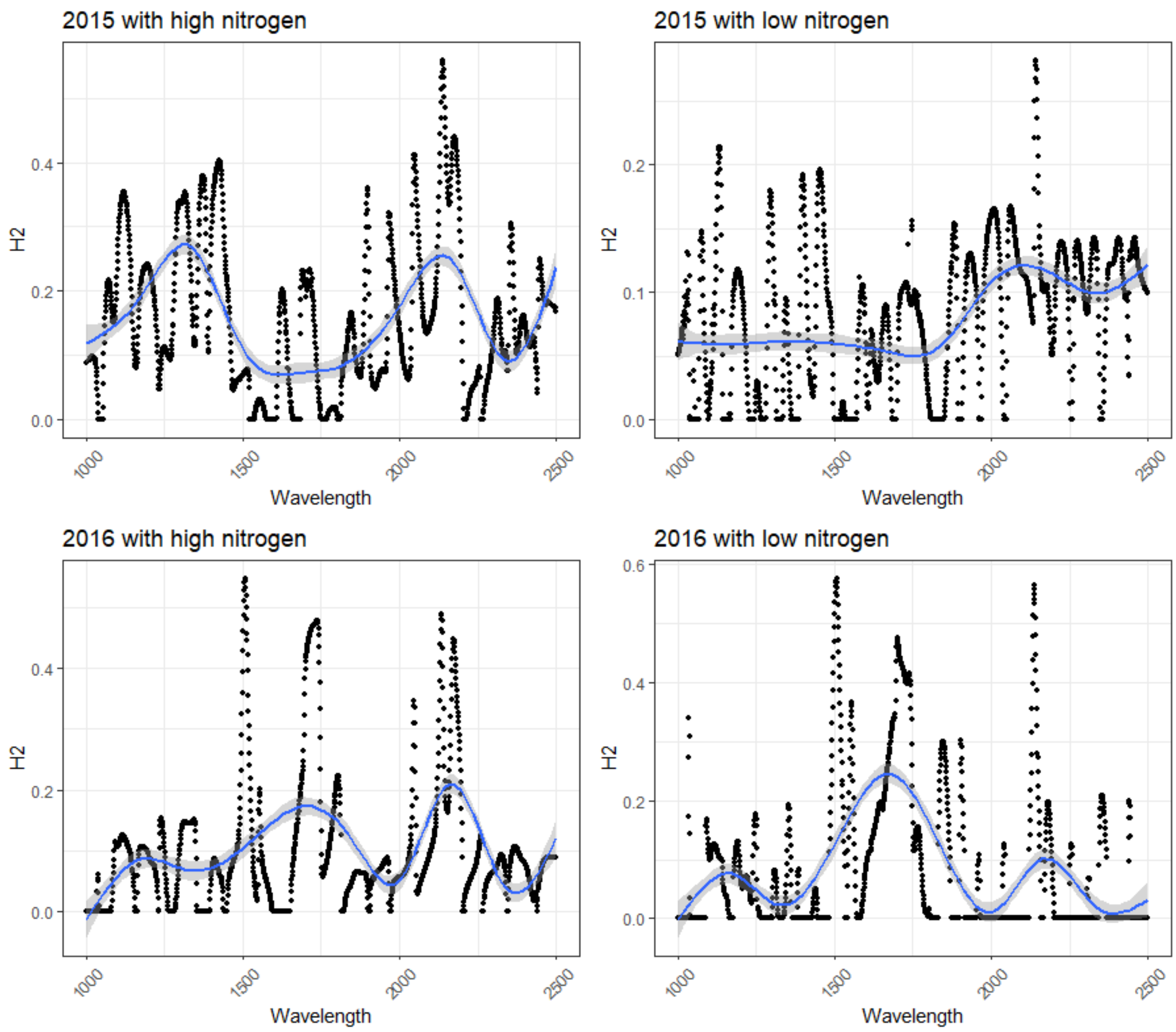

S1 Fig. Wavelength heritability after der2 pre-processing within each environment.

The blue lines represent the mean of heritability, performed using the geom_smooth option of the ggplot function of ggplot2 R package (Wickham H, 2016).
